# Effectiveness of Diverse Mesoporous Silica Nanoparticles as Potent Vehicles for the Drug L-DOPA

**DOI:** 10.3390/ma12193202

**Published:** 2019-09-30

**Authors:** Sumita Swar, Veronika Máková, Ivan Stibor

**Affiliations:** Department of Nanomaterials in Natural Science, Institute for Nanomaterials, Advanced Technologies and Innovation, Technical University of Liberec, Studentská 1402/2, 46117 Liberec, Czech Republic; dearsumita@gmail.com (S.S.); ivan.stibor@tul.cz (I.S.)

**Keywords:** mesoporous silica, nanoparticles, characterisation, electron microscopy, nitrogen adsorption, drug loading and release

## Abstract

Our study was focused on the synthesis of selective mesoporous silica nanoparticles (MSNs: MCM-41, MCM-48, SBA-15, PHTS, MCF) that are widely studied for drug delivery. The resulting mesoporous surfaces were conveniently prepared making use of verified synthetic procedures. The MSNs thus obtained were characterized by Brunauer-Emmett-Teller (BET) analysis and scanning electron microscopy (SEM). The selected MSNs with various pore diameters and morphologies were examined to evaluate the capability of L-DOPA drug loading and release. L-DOPA is a well-known drug for Parkinson’s disease. The L-DOPA drug loading and release profiles were measured by UV-VIS spectroscopy and SBA-15 was proved to be the most effective amongst all the different types of tested mesoporous silica materials as L-DOPA drug vehicle.

## 1. Introduction

The worldwide market for CNS therapeutics is steadily increasing, with the prognosis that in 2025 it could reach a value of 128.9 billion USD [1]. Among others, one cause of this rapid increase could be that the incidence of many CNS disorders increases exponentially after the age of 65 and the number of people in the world over 65 is increasing sharply. It takes longer to get a CNS drug to the market (12–16 years) compared with a non-CNS drug (10–12 years). The reason for this may lie in the complexity of the brain, the liability of CNS drugs to cause CNS side effects, and the requirement of CNS drugs to cross the blood-brain barrier (BBB) [2]. A major reason for the lack of progress in treating chronic neurodiseases is due to the presence of the BBB, a physical barrier between the CNS parenchyma and vasculature that plays a critical role in maintaining homeostasis within the CNS. Tight junctions exist between endothelial cells that inhibit paracellular diffusion of polar molecules, macromolecules and cells. These forces solute transport into the CNS to occur primarily across individual endothelial cells. Currently, 98% of small-molecule therapeutics and essentially 100% of large-molecule therapeutics, including monoclonal antibodies, proteins and gene therapies, cannot cross the BBB [3]. Numerous multidisciplinary-based strategies for transporting therapeutics from the blood into the brain through the blood-brain barrier have been proposed [3,4], including the use of receptor-mediated transcytosis in combination with different types of inorganic or organic nanoparticles (NPs) [3]. In 2013, Wiley et al. reported the use of gold NPs with transferrin in the delivery of a wide variety of therapeutics, some of which have already reached the clinical testing stage in humans [3,4,5]. Lamanna et al. designed, synthesised and characterised superparamagnetic iron oxide (SPIO) nanoparticles bearing different functional groups [6]. Progress in using iron oxide NPs for biological and biomedical applications [7,8] has advanced rapidly thanks to the tremendous work achieved in the synthesis and functionalization of these nanomaterials [9,10]. However, these NPs have some limitations in use such as stability in biological solutions at pH 7.4 close to the physiological blood. 

The design of nanoparticles for biomedical applications is still challenging [6]. Moreover, several problems related to targeted nanoparticles are always observed. These include agglomeration, distribution, transport efficiency, too-early or too-late degradation, cytotoxicity, biocompatibility etc. Particles with average hydrodynamic sizes of 10–100 nm are optimal for in vivo delivery. Due to the reasons mentioned above, very promising materials in these areas seem to be the mesoporous silica nanoparticles (MSNs). In general, the mesoporous materials are defined by IUPAC as materials with pore sizes between 2 to 50 nm [11]. These materials belong to the nanoporous material family having a pore size of the materials less than 100 nm. The microporous (pore size less than 2 nm) and macroporous (pore size more than 50 nm) materials also come under the classification of nanoporous materials. Mesoporous silica nanoparticles (MSNs) are among the best known and most widely used porous materials [11,12,13,14,15,16]. Thanks to their morphologies including high surface area, tunable pore sizes and large pore volumes that find these material’s diverse applications in catalysis, sorption, separations, sensing, optics and drug delivery [17]. The surfactant micelle-templated mesoporous silica materials are mainly classified as: mobile crystalline materials (MCM-41, MCM-48, MCM-50), Santa Barbara amorphous type materials (SBA-15, SBA-16), Michigan State University materials (MSU), Korean Advanced Institute of Science and Technology material (KIT-1, KIT-16), plugged hexagonal templated silica (PHTS), mesostructured cellular foam (MCF) and (FSM-16) [11,15].

The use of mesoporous material MCM-41 for a drug delivery system was firstly proposed in 2007 by Vallet-Regi et al. Biocompatible MSN-based controlled release systems have been demonstrated to be able to deliver different guest molecules (drugs) [14,15]. With the rapid development of silica-based drug delivery systems over the past decades, the use of pure mesoporous silica suffers from limitations such as targeted drug delivery mechanisms’ study, drug kinetics marker in pharmacological research, and track/evaluate the efficiency of the drug release in disease diagnosis and therapy [14]. Therefore, functionalized mesoporous silica materials with luminescence or magnetism have emerged with time [12]. The smart combination of different functional groups with MSNs has been investigated for the development of multifunctional medical platforms aiming simultaneous targeted delivery, fast diagnosis, and efficient therapy [12,18,19]. Very recently, the redox-responsive mesoporous organosilica nanoparticles containing disulfide bridges have been developed with higher efficacy for drug delivery system [20]. Therefore, more researches are being attracted to exploring new possibilities for MSN application in drug delivery.

The synthetic methods that were applied to produce the specific MSNs in this work are well verified by various other researches [11,12,15]. Our research was focused on the potential of using mesoporous nanoparticles for specifically L-DOPA drug loading and release. Mentioned nanoparticles have been studied and evaluated with the aim to find the most convenient combination of these nanoparticles and L-DOPA for needs Parkinson’s disease treatments.

## 2. Materials and Methods 

### 2.1. Materials 

Cetyltrimethylammonium bromide (99%, CTAB), tetraethyl orthosilicate (98%, TEOS), fumed silica powder (0.2–0.3 µm average particle size, SiO_2_), poly(ethylene glycol)-block-poly(propylene glycol)-block-poly(ethylene glycol)—P123 (Mw = 5800 g/mol), 1,12-dibromododecane (≥96%), *N*,*N*-dimethylhexadecylamine (≥95%, GC) and mesitylene (98%) were supplied by Merck (Darmstadt, Germany). Tetraethylammonium hydroxide (25% in water, TEAOH) and pyridine (99.5%) were purchased from ACROS Organics (Geel, Belgium). Ammonium fluoride (NH_4_F, 99.2%) was supplied by Lach:Ner (Neratovice, Czech Republic). Ethanol (99.9%, EtOH), methanol (99%, MeOH), ammonium hydroxide (NH_4_OH, 25%), hydrochloric acid (HCl, 35%) and sodium hydroxide (NaOH) were supplied by Penta (Prague, Czech Republic). Milli-Q water was used for nanoparticle synthesis and purification. 3-(3,4-Dihydroxyphenyl)-L-alanine (>98%, L-DOPA) was purchased from TCI EUROPE N.V. (Zwijndrecht, Belgium). Liquid nitrogen 5.0 (N_2_, 99.99% purity) was obtained from Linde (Liberec, Czech Republic).

### 2.2. Synthesis of Mesoporous Silica (SiO_2_) Nanoparticles (MSNs)

#### 2.2.1. MCM-41 (Spherical—S)

CTAB (3.75 g) was added to Milli-Q water (70.71 g) in a round bottom flask (250 mL) and stirred at 500 rpm/1 h/45 °C. Then NH_4_OH 25% solution (25.74 g) and EtOH (90 g) were added at 500 rpm/30 min. TEOS (7.05 g) was introduced dropwise into the stirring solution at 500 rpm for 3 h. The mixture was stirred at 300 rpm/12 h/25 °C. Finally, the mixture was filtrated under vacuum and thus obtained product was washed with distilled water and methanol (150 mL). The particles were dried at 90 °C/20 h and the post-treatment was achieved by calcination at 550 °C/5 h with a heating rate of 1°C/min in an ambient atmosphere.

#### 2.2.2. MCM-41 (Highly Ordered—HO)

NH_4_OH 22.7% solution (106.5 g) was mixed with Milli-Q water (116.5 g) of to form a homogeneous solution in a round bottom flask (250 mL). Subsequently, CTAB (1 g) was added at 500 rpm/45 °C until a homogeneous solution was obtained. When the solution reached to room temperature (r.t.), TEOS (4.67 g) was added and the mixture was stirred at 500 rpm/2 h/room temperature. The resulting precipitate was collected using vacuum filtration, washed with distilled water until neutralization and dried at 90 °C overnight. Finally, the product was calcined at 550 °C/6 h.

#### 2.2.3. MCM-48 

Milli-Q water (120 g) and NaOH (0.69 g) were added to Gemini 16-12-16 surfactant (5.8 g) in a round bottom flask (250 mL). The Gemini surfactant was prepared according to the procedure mentioned in the literature [10]. The solution was stirred at 700 rpm/room temperature until the surfactant was dissolved. Fumed silica (4 g) was added and the mixture was stirred at 700 rpm/2 h. The sealed flask was aged in an oven at 130 °C/3 days. Thereafter, the product was filtered, washed 4 times with deionized water (100 mL) and centrifuged at 10,000 rpm/5 min. Particles were heated in an oven at 130 °C/24 h. Finally, the product was recovered by vacuum filtration and further washed thrice with 150 mL distilled water. The obtained product was calcined at 550 °C/6 h.

#### 2.2.4. SBA-15

P123 (4 g) was added into the solution of Milli-Q water (130 g) and HCl 35% (21 mL) in a round bottom flask (250 mL). The solution was stirred at 800 rpm/3 h/room temperature. TEOS (8.53 g) was introduced and the mixture was stirred overnight at 45 °C. Ageing of the white precipitate was carried out at 80 °C/24 h, in the sealed flask without stirring. The product was collected by vacuum filtration, washed thrice with 50 ml of distilled water, then dried overnight at 80 °C and finally calcined at 550 °C/6 h.

#### 2.2.5. PHTS (Plugged Hexagonal Templated Silica)

P123 (4 g) was added into the solution of Milli-Q water (130 g) and HCl 35% (21 mL) in a round bottom flask (250 mL). The solution was stirred at 800 rpm/3 h/room temperature. Then TEOS (14.93 g) was added into the mixture and the mixture was continuously stirred at 60 °C/overnight. The product aging was carried out for at 80 °C/24 h, then collected by vacuum filtration, washed thrice with 50 mL of distilled water, dried overnight at 80 °C and finally calcined at 550 °C/6 h. 

#### 2.2.6. MCF (Mesostructured Cellular Foam)

P123 (4 g) was added into the solution of Milli-Q water (130 g) and HCl 35% (21 mL) in a round bottom flask (250 mL) stirred at 700 rpm/room temperature until the surfactant was dissolved. NH_4_F (47 mg) and mesitylene (4.6 mL) were introduced into the flask. The temperature was raised to 40 °C and stirring was continued at 800 rpm/1 h. Then, TEOS (8.53 g) was added and stirred at 40 °C/20 h. The mixture was transferred to an autoclave and kept at 100 °C/24 h. The product was collected by vacuum filtration, washed thrice with 50 mL of water and finally calcined at 550 °C/6 h. Yields of all the prepared samples are summarised in the Table 1.

### 2.3. Analyses

#### 2.3.1. Scanning Electron Microscopy (SEM)

Size distribution and shape homogeneity of MSNs were examined by SEM (ZEISS, Jena, Germany) images. Samples were prepared by taking small quantities of MSNs dispersed in distilled water. Then the solution was dropped on the carbon-coated copper grid and dried under vacuum to ensure the complete removal of the solvent. MSNs were sputtered with 2 nm platinum layer, subsequently were viewed as secondary electron images (2 kV).

#### 2.3.2. Nitrogen Adsorption

The gas sorption analyser (Autosorb® iQ, Quantachrome Instruments, Ashland, OR, USA) was employed to examine the surface areas and pore size distributions of prepared MSNs. The surface areas and pore size distributions were calculated using ASiQwin software (version for Windows XP) based on adsorption-desorption isotherms. The pristine samples were degassed at 300 °C/3 h. Then N_2_ adsorption and desorption isotherms were measured at the temperature of −196 °C. Multipoint BET (Brunauer-Emmett-Teller) analysis was applied for the total surface area calculation. Models of DFT (Density Functional Theory) were used to determine pore size distribution and also compared to relatively old BJH (Barret-Joyner-Halenda) model. 

### 2.4. Drug Loading on and Releasing from MSNs

#### 2.4.1. Preparation of L-DOPA Solution

Milli-Q water (50 mL) was added to L-DOPA (50 mg) in an Erlenmeyer flask (100 mL) covered with aluminium foil and sonicated for 1 h/18 °C for complete dissolution. The solution was used for drug loading and calibration.

#### 2.4.2. L-DOPA Loading

The drug L-DOPA was loaded by soaking silica (10 mg) in 1 mg/mL of L-DOPA solution (in Milli-Q water) for 2 h, 4 h, 6 h, 15 h and 24 h. The drug-loaded MSNs were stored into the refrigerator (4 °C) covered with aluminium foil as L-DOPA solution is sensitive towards heat and light. The drug-loaded samples were centrifuged at 10,000 rpm/10 min and the clear solution above the precipitate was collected for examining the loading profiles each of the samples. The L-DOPA loaded solid samples were collected after filtration and dried in the desiccator.

#### 2.4.3. L-DOPA Release

L-DOPA loaded samples (100 mg) were dispersed in a phosphate buffer solution (10 mL) with pH 7.2 (PBS, K_2_HPO_4_ and KH_2_PO_4_) and kept at 37 °C using an incubator in order to simulate the body temperature during 15 min, 30 min, 1 h, 2 h and 3 h. A higher amount of drug was expected to be released into the intestine at pH range 7–8 [20]. Monitoring of drug loading and releasing was accomplished by UV spectrophotometry.

Both the drug loading and releasing profiles for each of the samples were determined by monitoring the absorbance change using the UV-VIS spectrophotometer (DR 6000, HACH^®^, Prague, Czech Republic, wavelength range: 190–1100 nm, scanning speed: 900 nm/min). The calibration curve (for drug loading profile) was prepared by measuring the absorbance at various suitable concentrations of L-DOPA solution in water and absorption peaks were recorded at 290 nm for L-DOPA. Similarly, for drug releasing profile, the calibration curve was also obtained for L-DOPA solution in PBS.

### 2.5. Statistical Analyses

The experiments were performed three times and standard deviation (SD) was calculated using Excel (Office Professional Plus 2016, Microsoft). Student’s *t* test (α = 0.05) was used to evaluate whether the difference was statistically significant.

## 3. Results and Discussion

### 3.1. Synthesis of Mesoporous Silica Nanoparticles 

**Figure 1:**
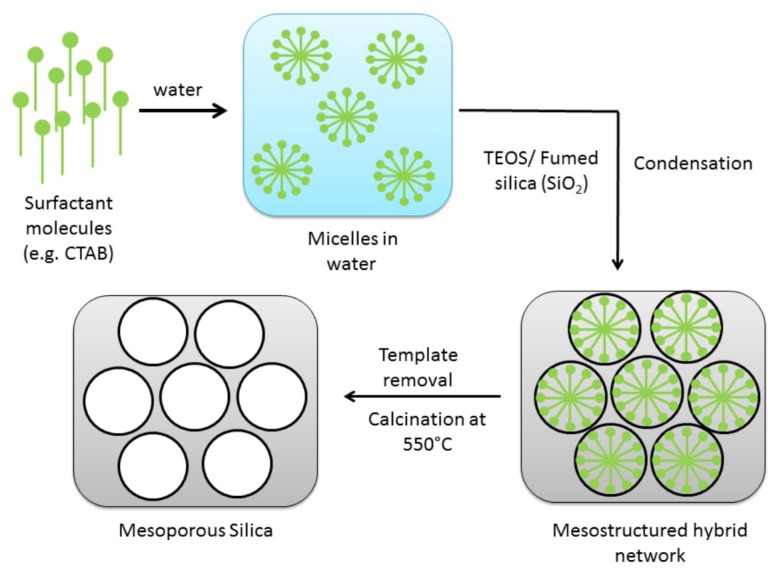
Overview of a synthetic approach to the mesoporous silica formation.

The schematic overview of the synthetic approach to mesoporous silica materials is shown in Figure 1. The synthesis of templated mesoporous silica follows a few steps: dissolution of template molecules (CTAB or P123) in the solvent (usually water); addition of the silica source (TEOS or fumed silica); stirring at required conditions to allow the hydrolysis and pre-condensation; recovery of the product and the final stage of template removal by calcination [11]. Hydrolysis may occur both in acidic or basic medium [13].

Soft templating, including micelle templating, offers an alternative facile and environmentally friendly approach for MSN preparation. The structural transformation of amphiphilic surfactant organizations can be understood by the surfactant packing factor/parameter, g = V/l.a_0_, where V is the volume of the hydrophobic chains in surfactant, l is the surfactant chain length, and a_0_ is the effective area of the hydrophilic head group of the surfactant at the interface [17]. Generally, surfactants with lower critical micelle concentration (CMC) are more favoured to obtain ordered structure. According to the classical micelle chemistry, above a critical value, g-packing factor increases and therefore, mesophase transitions occur. When g < 1/3, particles tend to form Pm3n cubic structures and mixed 3D hexagonal and cubic structures; when 1/3 < g <1/2, particles tend to form p6mm hexagonal structures; when 1/2 < 2/3, particle tend to form Ia3d cubic structures; when g ~ 1, lamellar structures are favoured [11,17].

### 3.2. Characterisation Methods

#### 3.2.1. Morphology of the Samples

**Figure 2:**
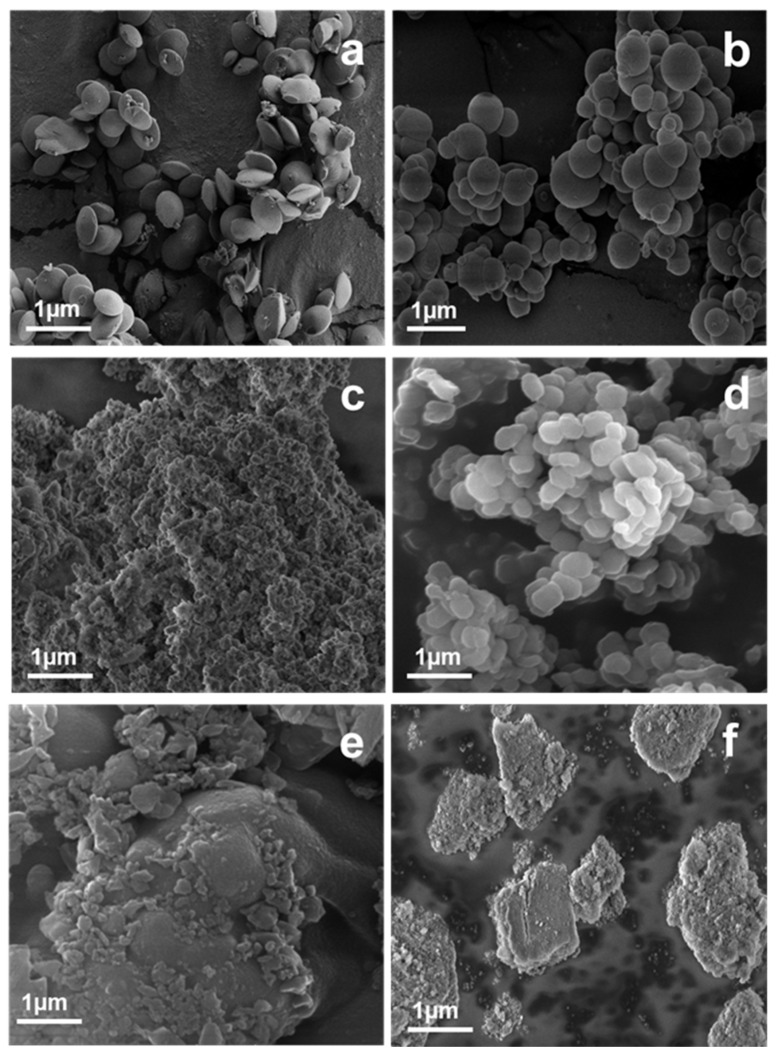
SEM images of MSNs: MCM-41(HO) (**a**); MCM-41(S) (**b**); MCM-48 (**c**); SBA-15 (**d**); PHTS (**e**) and MCF (**f**).

The SEM micrographs in Figure 2a–f reveal that the obtained samples have different morphologies, with various shapes and sizes (see Table 2). MCM-41(HO) and MCM-41(S) showed highly ordered conical disc-shaped and spherical nanosized particles, respectively, with a polydispersity of particle size (Figure 2a,b) [11,16,21]. 

MCM-41(HO) exhibited more uniform nanoparticles with sizes between 400–600 nm. On the contrary to this, the particle sizes of MCM-41(S) were observed in the range of 200–900 nm. Figure 2c,e,f (MCM-48, PHTS and MCF, respectively) show the agglomeration of the MSNs into clusters (>1 μm) where the smaller particles are also clearly visible. SBA-15 (Figure 2d) revealed comparatively uniform bagel-shaped particles about the size 300–500 nm [21]. The morphology of mesopores in the silica particles could not be evaluated by SEM. The nature of the porous structures was examined by BET analysis.

#### 3.2.2. Surface area and Porosity of the Prepared Samples

**Figure 3:**
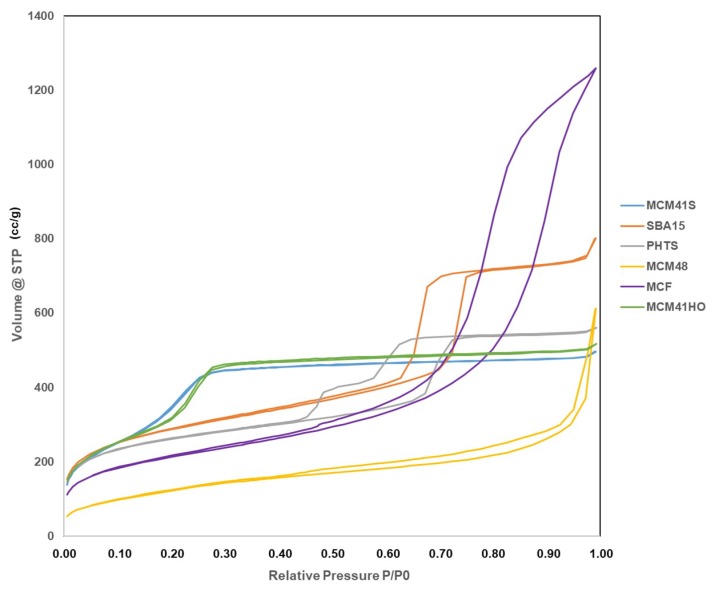
Nitrogen adsorption-desorption isotherms at −196 °C for MCM-41(S); MCM-41(HO); MCM-48; SBA-15; PHTS and MCF.

Figure 3 shows nitrogen adsorption and desorption isotherms of the six different types MSNs: MCM-41(S); MCM-41(HO); MCM-48; SBA-15; PHTS and MCF. All the samples can be classified as type IV isotherms according to the IUPAC classification and that is typical to the mesoporous silica materials [22]. MCM-41(S) and MCM-41(HO) exhibited similar N_2_ sorption isotherms where an increase in adsorption took place above P/P_0_ = 0.2, suggesting the capillary condensation of N_2_ within the pores and confirmed the presence of mesopores [21]. MCM-48 demonstrated adsorption isotherm at a higher relative pressure (P/P_0_ > 0.9) that corresponds to N_2_ condensation in the interparticle voids and also reflects the small size of mesoporous material [23]. SBA-15 exhibited H1 hysteresis loop starting from P/P_0_ = 0.6, that is characteristic of SBA-15 with highly ordered pores [23]. The adsorption-desorption behaviour of PHTS was consistent with a structure comprising both open and blocked cylindrical mesopores [22]. PHTS showed two-step desorption where the first desorption step was similar to that of SBA-15, indicating the open mesopores. The second desorption step of PHTS was attributed to the plugs (NPs) within the mesoporous. For the plugged pores, desorption was delayed resulting in second desorption [11]. The hysteresis loop was appeared at P/P_0_ = 0.4–0.75. The MCF N_2_ sorption isotherm with characteristic long H2 hysteresis loop was in a good agreement with other article [24].

**Figure 4:**
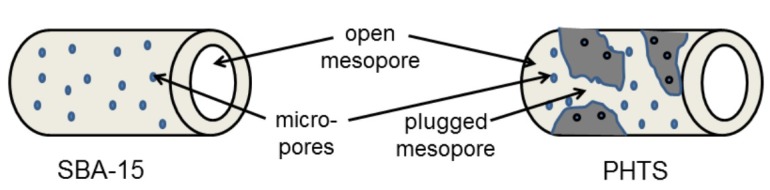
Schematic representation of the pore structure: SBA-15 and PHTS.

Figure 4 depicts the difference between usual mesoporous material such as SBA-15 and PHTS with plugged pores. PHTS mesopores are narrowed by nanoparticles (plugs) to create inkbottle-like sections. On the contrary, SBA-15 has open mesopores [11]. Usually, small micropores are also exhibited on the walls of MSNs.

The characteristics of calcined mesoporous silica materials were analysed by BET for examining their specific surface areas. The pore size distributions were determined by the BJH model that was further verified by DFT model. All the results are summarised in Table 3. The previously widely used BJH model is no longer recommended for such applications in micro-mesoporous materials examination as it can significantly underestimate the pore size for narrow mesopores (for pore diameter smaller than 10 nm the pore size may be underestimated even by 30%). However, BJH modelling was used to compare the synthesised materials with the specifications found in the literature [11]. All the MCM-41 samples exhibited high specific surface area lying between 700–1120 m^2^/g. The pore diameters were in the range of ~2–4 nm, although their morphologies were different. MCM-48 showed relatively low surface area (470 m^2^/g) compared to other MSNs, although the pore diameter is similar to the MCM-41(S) sample. The specific surface area of SBA-15 was 1020 m^2^/g with larger pore diameter (~8 nm) compared to MCM series. PHTS also displayed high surface area with 940 m^2^/g and the pore diameter of open pores was between 5–7.5 nm, whereas the plugged pores were less than 2 nm. In this case, the DFT was considered a more reliable method than BJH as there was a significant difference in adsorption-desorption pore diameter for plugged pores. MCF displayed the highest pore size of 15–10 nm with 760 m^2^/g surface area. SBA-15 and MCF exhibited comparatively higher pore volumes (2.34, 1.17 and surface area 1.88 cm^3^/g respectively). On the other hand, MCM-41(S), MCM-41(HO), MCM-48 and PHTS possessed the pore volumes ~0.7–0.8 and surface area only 88 cm^3^/g.

For all the synthesized MSNs, N_2_ sorption isotherms, BET and BJH (verified by DFT) data regarding pore sizes, surface areas and pore volumes were compatible with the verified once given in the literature [11]. Therefore, the pore structures (morphologies) are suggested to be the same as demonstrated in various literature mentioned earlier [15,17]. 

**Figure 5:**
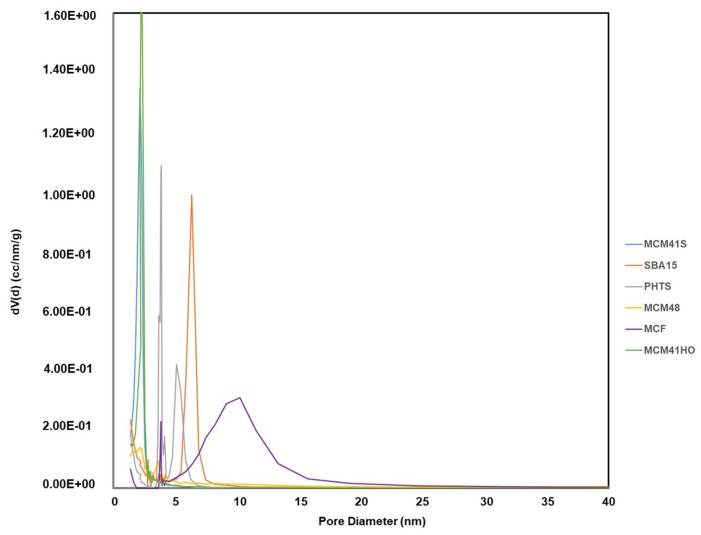
Pore size distribution of the prepared nanoparticles.

The morphological properties such as high surface area, large pore volume and the narrow particle size distribution are well suited for the application of nano-encapsulation in drug delivery systems. Moreover, different types of prepared mesoporous silica allowed us for precise comparison of their properties (high polydispersity in particle size, different porosity, particle morphology and pore structure) for L-DOPA drug release [25]. For the evaluation of L-DOPA delivery profile, MSNs with same pore sizes but different morphologies (MCM-41(S); MCM-41(HO)) and MSNs with various pore sizes (MCM-48, SBA-15, PHTS and MCF), illustrated in Figure 5, were chosen to understand the effect of pore size along with particle morphology on drug loading and release profile.

### 3.3. L-DOPA Loading and Release 

**Figure 6:**
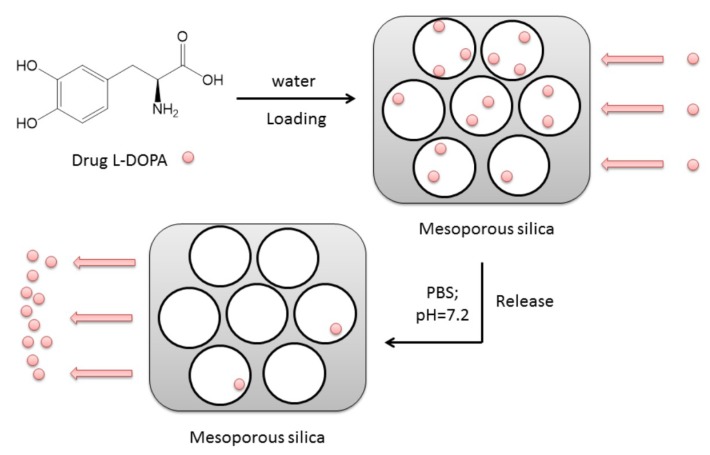
The illustration for L-DOPA drug loading to and release from mesoporous silica.

L-DOPA is an amino acid that is made and used as part of the normal human body as well as some animals and plants. It can cross the protective blood-brain barrier, unlike dopamine. Therefore, L-DOPA increases dopamine concentration for the common Parkinson disease treatment. This treatment was proven clinically by Nicholson and his group [26]. MSNs were widely explored for drug delivery using various types of drugs, whereas L-DOPA was not given any importance to having MSNs as the potential carriers [25,27]. In our research, the potential of using MSNs for specifically L-DOPA drug loading and release has been studied and evaluated. The illustration of the whole process for drug loading and release is given in Figure 6. 

The calculations for loading and release of drugs using mesoporous particles have been widely discussed in many research works [28,29]. 

#### 3.3.1. L-DOPA Drug Loading

**Figure 7:**
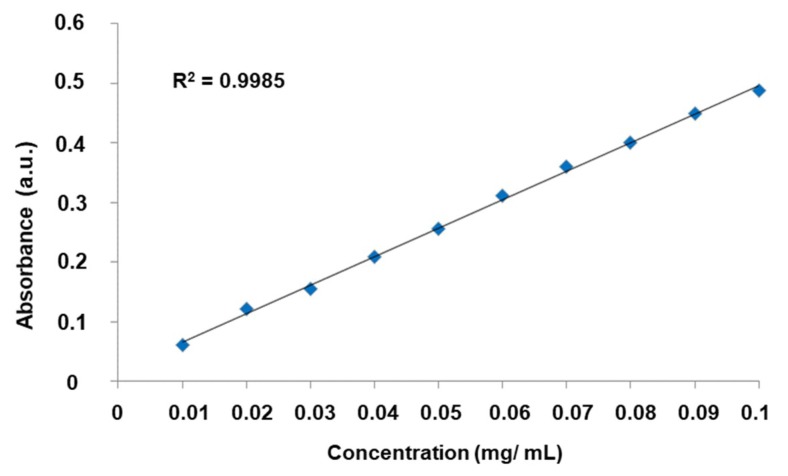
The standard calibration curve of L-DOPA in water.

L-DOPA drug loading was monitored by UV-VIS spectrophotometer and the calibration curve see Figure 7 was plotted to calculate the concentration change with respect to time using the absorbance spectra for evaluating the L-DOPA loading.

**Figure 8:**
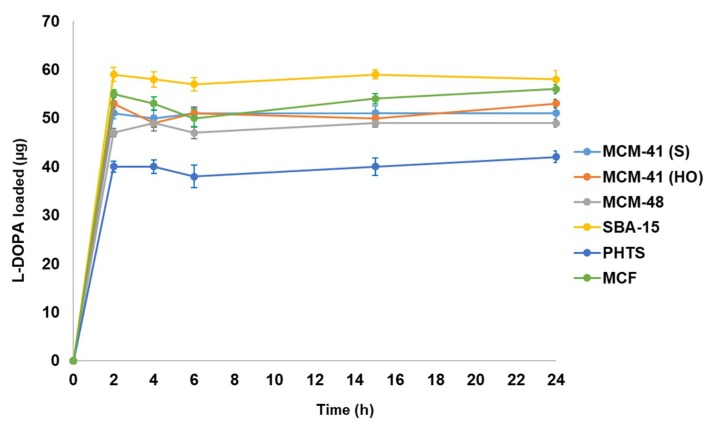
The L-DOPA loading profiles for all six types of mesoporous silica particles.

The loading profiles of L-DOPA for MSNs are presented in Figure 8. We expected to observe the differences in drug loading amount as the mentioned MSNs had different surface morphologies although the pore sizes were similar. 

The curves show the drug loading (in µg) per 10 mg of the prepared mesoporous silica materials. Maximum loading was allowed by SBA-15 (59 µg) amongst all the mesoporous silica materials. SBA-15 is widely used for drug delivery and the obtained comparative results also support SBA-15 as the best mesoporous silica material. MCM-41(S), MCM-41(HO) and MCM-48 showed the narrow difference in loaded drug (51 µg, 53 µg and 49 µg respectively) as the pore diameters of all MCM materials were found to be similar. Highly ordered MCM-41(HO) exhibited more loading compared to other MCM series materials due to uniformity of porosity as well as surface morphology. The MCM-41(S) had spherical morphology, but particle sizes were less uniform than MCM-41(HO), therefore loading was less than MCM-41(HO). Due to large pore sizes of MCF, the loading performance of it (56 µg) was very close to SBA-15 and PHTS demonstrated minimum loading (42 µg), probably due to the presence of plugged pores. The L-DOPA loading was observed to be fast (2 h). The loadings were not continued more than 24 h as L-DOPA aqueous solution is very sensitive to light and heat. Moreover, the solution turns black as a result of degradation in solution [30]. The amounts of L-DOPA (wt.%) drug loaded in different samples are summarised in Table 4.

#### 3.3.2. L-DOPA Drug Release

**Figure 9:**
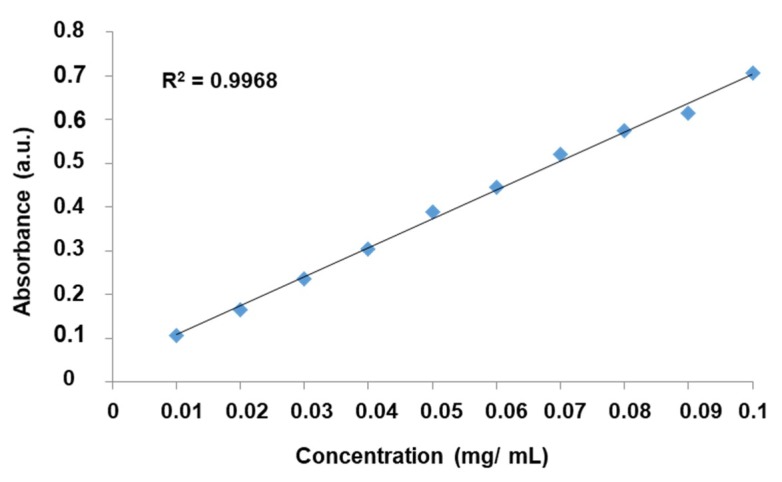
The standard calibration curve of L-DOPA in PBS.

Like L-DOPA drug loading, L-DOPA drug release in PBS was also monitored by UV-VIS spectrophotometer. The calibration curve (Figure 9) was plotted using the absorbance spectra to measure the released drug concentration directly with respect to time for L-DOPA in PBS at pH 7.2.

**Figure 10:**
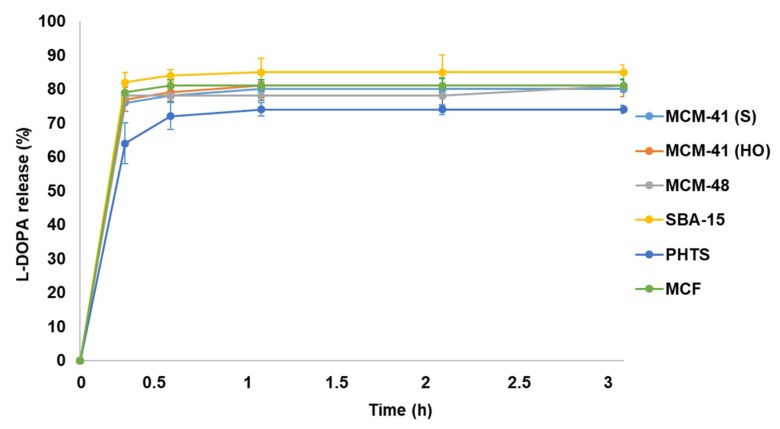
The L-DOPA drug release profiles for all six types of mesoporous silica particles.

The absorbance spectra recorded during the release of L-DOPA drug in different time intervals were used in order to prepare a plot indicating the release profile. The release profiles are depicted in Figure 10. 

The results established that the release is sustained rather than prolonged [27]. The release profiles of all tested materials were quite similar. SBA-15 with high surface area and pore volume (pore diameter ~7.6 nm) achieved highest release amount with ~85% in 1h whereas, PHTS revealed the lowest release (~74%). In MCM series, MCM-41 (HO) showed more drug release capacity (~81%) than others and all the results were compatible with the drug loading profiles. MCM-48 released ~78% of loaded L-DOPA, but MCM-41 (S) released ~80%. MCF having comparatively large pores (~12 nm) achieved ~83% drug release. 

The comparative studies of drug loading and release revealed that surface morphologies, specific surface area, pore volumes and pore diameters played an important role in drug loading and release profiles of different MSNs. Amongst all the tested mesoporous silica materials, SBA-15 was found to be the best for L-DOPA drug loading and release. The analyses of drug loading and release profiles revealed that MSNs can be used for effective L-DOPA drug delivery choosing suitable mesoporous silica.

### 3.4. Up and Coming Outcomes Related to Biocompatibility Assessments

Numerous research papers are focused on the biocompatibility of various types MSNs [31,32]. Moreover, the chemo-physical properties of particles including their size, shape, surface area, structure and the route of drug administration have proved to play significant roles in their biocompatibility. For example, small MSNs (50 nm) exhibited effective drug delivery from the aspect of cellular uptake. On the contrary, large submicron particles (1220 nm) showed less cytotoxicity than nanoparticles (190 nm and 420 nm) [32]. Unfortunately, the attempts are limited by less understanding of particle interactions with cells in circulation. Current knowledge related to the biocompatibility for MSNs does not match with the rapid pace of research and sometimes is misleading [31]. With the rapid development of biomaterials, the original concept of “biocompatibility” has widely deviated. The biocompatibility may include all damaging as well as beneficial biological effects caused by MSNs [31,33,34]. Therefore, an appropriate protocol for an effective biocompatibility evaluation should be always chosen concerning all the important parameters, which are necessary for safety evaluation, mainly in case, of drug delivery systems.

## 4. Conclusions

Six different mesoporous silica materials were successfully prepared. The SEM and BET techniques were applied to evaluate the morphology and particle characteristics (surface areas, pore volumes and pore size distributions) of the prepared materials. A comparative study focused on the efficiency of L-DOPA drug loading and release among all the prepared mesoporous silica samples, analysed via UV-VIS absorption spectroscopy, confirmed SBA-15 as the most promising material for further experiments related to the kinetics of L-DOPA drug delivery system. We assume that the pore size of MSNs plays much more important role than their surface morphologies as all the MCM-41 (S/HO) samples, with nearly similar pore size, demonstrated almost same delivery profiles, although their surface morphologies were very different. The successful L-DOPA loading is highly encouraging for the continuation of further studies on in vitro and in vivo biocompatibility for evaluating the potential of clinical application.

## Figures and Tables

**Table 1:** Yields of all the synthetized particles.

Sample	Yield (g)	Yield (%)
**MCM-41(S)**	2 ± 0.4	~28
**MCM-41(HO)**	0.9 ± 0.1	~20
**MCM-48**	3 ± 0.3	~75
**SBA-15**	2.8 ± 0.2	~32
**PHTS**	4.2 ± 0.7	~28
**MCF**	2.3 ± 0.5	~27

**Table 2:** The range of particle size and geometry of the particles obtained via SEM.

Sample	Geometry	Particle Size (nm)
**MCM-41(S)**	spheres	200–900
**MCM-41(HO)**	cone discs	400–600
**MCM-48**	agglomerated	>1000
**SBA-15**	bagel-shaped (short rods)	300–500
**PHTS**	agglomerated	>1000
**MCF**	agglomerated	>1000

**Table 3:** Calcined MSN samples evaluated via N_2_ adsorption.

Sample	BET Specific Surface Area (m^2^/g)	Pore Volume (cm^3^/g) [DFT model]	Pore Diameter (nm)		
			DFT Model	BJH Model	
				Adsorption Branch	Desorption Branch
**MCM-41(S)**	880	0.72	3.18	2.12	2.14
**MCM-41(HO)**	1120	0.75	3.30	2.25	2.26
**MCM-48**	470	0.77	3.18	2.12	2.22
**SBA-15**	1020	1.17	7.59	8.97	6.33
**PHTS**	940	0.83	5.06; 1.64	7.12; 0.89	5.23; 3.87
**MCF**	760	1.88	11.68	15.43	10.19

**Table 4:** L-DOPA loaded within the samples.

Sample	MCM-41 (S)	MCM-41 (HO)	MCM-48	SBA-15	PHTS	MCF
**L-DOPA loaded (wt.%)**	5.1 ± 0.3	5.3 ± 0.5	4.9 ± 0.3	5.9 ± 0.3	4.2 ± 0.6	5.6 ± 0.2

## References

[1] Central Nervous System (CNS) Therapeutic Market Report, 2018–2025. https://www.grandviewresearch.com/industry-analysis/central-nervous-system-cns-therapeutic-market.

[2] Wilhelm I., Krizbai I.A. (2014). In Vitro Models of the Blood–Brain Barrier for the Study of Drug Delivery to the Brain. Mol. Pharm..

[3] Wiley D.T., Webster P., Gale A., Davis M.E. (2013). Transcytosis and Brain Uptake of Transferrin-Containing Nanoparticles by Tuning Avidity to Transferrin Receptor. Proc. Natl. Acad. Sci. USA.

[4] Kamaly N., Xiao Z., Valencia P.M., Radovic-Moreno A.F., Farokhzad O.C. (2012). Targeted Polymeric Therapeutic Nanoparticles: Design, Development and Clinical Translation. Chem. Soc. Rev..

[5] Davis M.E., Zuckerman J.E., Choi C.H.J., Seligson D., Tolcher A., Alabi C.A., Yen Y., Heidel J.D., Ribas A. (2010). Evidence of RNAi in Humans from Systemically Administered SiRNA via Targeted Nanoparticles. Nature.

[6] Lamanna G., Kueny-Stotz M., Mamlouk-Chaouachi H., Ghobril C., Basly B., Bertin A., Miladi I., Billotey C., Pourroy G., Begin-Colin S. (2011). Dendronized Iron Oxide Nanoparticles for Multimodal Imaging. Biomaterials.

[7] Fang C., Zhang M. (2009). Multifunctional Magnetic Nanoparticles for Medical Imaging Applications. J. Mater. Chem..

[8] Qiao R., Yang C., Gao M. (2009). Superparamagnetic Iron Oxide Nanoparticles: From Preparations to in Vivo MRI Applications. J. Mater. Chem..

[9] Berry C.C. (2009). Progress in Functionalization of Magnetic Nanoparticles for Applications in Biomedicine. J. Phys. D Appl. Phys..

[10] Thanh N.T.K., Green L.A.W. (2010). Functionalisation of Nanoparticles for Biomedical Applications. Nano Today.

[11] Meynen V., Cool P., Vansant E.F. (2009). Verified Syntheses of Mesoporous Materials. Microporous Mesoporous Mater..

[12] Yang P., Gai S., Lin J. (2012). Functionalized Mesoporous Silica Materials for Controlled Drug Delivery. Chem. Soc. Rev..

[13] Brinker C.J. (1988). Hydrolysis and Condensation of Silicates: Effects on Structure. J. Non-Cryst. Solids.

[14] Wang Y., Zhao Q., Han N., Bai L., Li J., Liu J., Che E., Hu L., Zhang Q., Jiang T. (2015). Mesoporous Silica Nanoparticles in Drug Delivery and Biomedical Applications. Nanomed. Nanotechnol. Biol. Med..

[15] Douroumis D., Onyesom I., Maniruzzaman M., Mitchell J. (2013). Mesoporous Silica Nanoparticles in Nanotechnology. Crit. Rev. Biotechnol..

[16] Deng X., Chen K., Tüysüz H. (2017). Protocol for the Nanocasting Method: Preparation of Ordered Mesoporous Metal Oxides. Chem. Mater..

[17] Wu S.H., Mou C.Y., Lin H.P. (2013). Synthesis of Mesoporous Silica Nanoparticles. Chem. Soc. Rev..

[18] He Y., Luo L., Liang S., Long M., Xu H. (2017). Amino-Functionalized Mesoporous Silica Nanoparticles as Efficient Carriers for Anticancer Drug Delivery. J. Biomater. Appl..

[19] Xu X., Wu C., Bai A., Liu X., Lv H., Liu Y. (2017). Folate-Functionalized Mesoporous Silica Nanoparticles as a Liver Tumor-Targeted Drug Delivery System to Improve the Antitumor Effect of Paclitaxel. J. Nanomater..

[20] Maggini L., Cabrera I., Ruiz-Carretero A., Prasetyanto E.A., Robinet E., Cola L.D. (2016). Breakable Mesoporous Silica Nanoparticles for Targeted Drug Delivery. Nanoscale.

[21] Zukal A., Šiklová H., Čejka J., Thommes M. (2007). Preparation of MCM-41 Silica Using the Cationic Surfactant Blend. Adsorption.

[22] Vazquez N.I., Gonzalez Z., Ferrari B., Castro Y. (2017). Synthesis of Mesoporous Silica Nanoparticles by Sol–Gel as Nanocontainer for Future Drug Delivery Applications. Bol. Soc. Esp. Ceram. Vidr..

[23] Yu J., Shen L., Cao Y., Lu G. (2016). Preparation of Pd-Diimine@SBA-15 and Its Catalytic Performance for the Suzuki Coupling Reaction. Catalysts.

[24] Hermida L., Abdullah A.Z., Mohamed A.R. (2013). Synthesis and Characterization of Mesostructured Cellular Foam (MCF) Silica Loaded with Nickel Nanoparticles as a Novel Catalyst. Mater. Sci. Appl..

[25] Rahmani S., Durand J.O., Charnay C., Lichon L., Férid M., Garcia M., Gary-Bobo M. (2017). Synthesis of Mesoporous Silica Nanoparticles and Nanorods: Application to Doxorubicin Delivery. Solid State Sci..

[26] Nicholson G., Pereira A.C., Hall G.M. (2002). Parkinson’s Disease and Anaesthesia. Br. J. Anaesth..

[27] Sevimli F., Yılmaz A. (2012). Surface Functionalization of SBA-15 Particles for Amoxicillin Delivery. Microporous Mesoporous Mater..

[28] Jangra S., Girotra P., Chhokar V., Tomer V.K., Sharma A.K., Duhan S. (2016). In-Vitro Drug Release Kinetics Studies of Mesoporous SBA-15-Azathioprine Composite. J. Porous Mater..

[29] Jangra S., Duhan S., Goyat M.S., Chhokar V., Singh S., Manuja A. (2017). Influence of Functionalized Mesoporous Silica in Controlling Azathioprine Drug Release and Cytotoxicity Properties. Mater. Res. Innov..

[30] Pulikkalpura H., Kurup R., Mathew P.J., Baby S. (2015). Levodopa in *Mucuna pruriens* and Its Degradation. Sci. Rep..

[31] Behzadi S., Serpooshan V., Tao W., Hamaly M.A., Alkawareek M.Y., Dreaden E.C., Brown D., Alkilany A.M., Farokhzad O.C., Mahmoudi M. (2017). Cellular Uptake of Nanoparticles: Journey inside the Cell. Chem. Soc. Rev..

[32] Tang F., Li L., Chen D. (2012). Mesoporous Silica Nanoparticles: Synthesis, Biocompatibility and Drug Delivery. Adv. Mater. Weinh..

[33] Zhao Y., Sun X., Zhang G., Trewyn B.G., Slowing I.I., Lin V.S.Y. (2011). Interaction of Mesoporous Silica Nanoparticles with Human Red Blood Cell Membranes: Size and Surface Effects. ACS Nano.

[34] Narayan R., Nayak U.Y., Raichur A.M., Garg S. (2018). Mesoporous Silica Nanoparticles: A Comprehensive Review on Synthesis and Recent Advances. Pharmaceutics.

